# Characterisation of unessential genes required for survival under conditions of DNA stress

**DOI:** 10.1186/s43141-020-00025-x

**Published:** 2020-05-06

**Authors:** Hassan Ahmed Ezzat, Clive Price

**Affiliations:** Division of Biomedical and Life Sciences, Faculty of Health and Medicine, Lancaster University, Lancaster, UK

**Keywords:** *Schizosaccharomyces pombe*, TopBP1, Eukaryotic DNA replication, DNA Fork Stalling

## Abstract

**Background:**

Genomic instability is a hallmark of cancer. Cancer progression depends on the development and amplification of mutations that alter the cellular response to threats to the genome. This can lead to DNA replication stress and the potential loss of genetic integrity of the newly formed cells. This study utilised fission yeast to map the interactions occurring in some of the most crucial pathways in both DNA replication and checkpoint monitoring involving Rad4, the *Schizosaccharomyces pombe* (*S. pombe*) TopBP1 homologue. We have modelled conditions of replication stress in the genetically tractable fission yeast, *S. pombe* using the hypomorphic *rad4-116* allele. Synthetic genetic analysis was used to identify processes required for cell survival under conditions of DNA replication stress. With the aim of mapping the genetic interactions of *rad4* and its mutant allele, *rad4-116*, several genes that could have an interaction with *rad4* during replication stress have emerged as attractive.

**Results:**

Interactions with genes involved in chromatin remodelling, such as *hip1*, and replication fork stalling resolution, such as *mrc1*, *swi1* and *swi3* were explored and confirmed. The interactions of Rad4 with each of the genes provided separate and distinct tumour formation pathways, as evident in the synthetically lethal interactions. Even within the same complex, *rad4-116* double mutants behaved differently proving that Rad4 interacts at different levels and functions with the same proteins.

**Conclusion:**

Results from this study provide a novel view of the *rad4* interactions, the association of Rad4 with the replisome. The study also provides the groundwork on a theoretical and practical level for the exploration and separation of interactions of TopBP1 with the histone chaperone family and the replisome.

## Background

Across all cell types, from the simplest bacteria to the most complex multicellular organisms, precise coordination of DNA replication with chromosome segregation during the cell division cycle is essential to ensure that both daughter cells inherit a complete and intact complement of genetic material. The relatively large genomes of eukaryotic cells are replicated from multiple replication origins on multiple chromosomes. DNA replication per se requires the activity of a number of DNA polymerases, the initial activity of which is confined to these origins [1]. The cell division cycle process has been linked to a wide variety of physiologic processes such as embryonic development and cell growth and pathologic processes such as cancer and Alzheimer’s disease [2]. This occurs as errors or defects in genes which play a role in DNA replication, DNA repair, or checkpoint response have been highlighted to play a key role in the predisposition to cancer [3, 4]. As a regular event occurring in cancer, replication stress can be identified as the stalling or slowing of replication forks causing any alteration in the regular replication process such as the unwinding of DNA through decoupling of replicative helicases and polymerases [5]. It can be viewed as an important event that is common across all cancer types to the point that it has been proposed as a hallmark of cancer [6].

To be able to pinpoint the origins, origins are bound by the origin recognition complex (ORC) which is a heterohexamer constituted of subunits named ORC 1, 2, 3, 4 5 and 6 with ORC1 binding being an early event in the regulation of replication [7, 8]. ORC1 and ORC6 bind relatively loosely to the origin while ORCs 2 to 5 bind strongly to the origin. The ORC complex then recruits CDC6 and CDT1 to form the PreRC which recruits the heterohexamer minichromosome maintenance complex (MCM). MCM is composed of 6 subunits MCM2-7 and promotes the recruitment of another MCM2-7 complex forming a double hexamer of MCM2-7s on chromatin during the process of replication licensing [9–11]. Then, cyclin E-bound CDK2 and DDK promote the recruitment of the CDC45-MCM2-7-GINS complex (CMG) which acts to separate the MCM2-7 hexamers, thereby, activating their helicase activity [12]. These structures are called pre-initiation complexes (PreICs), at which the replicative polymerases are now recruited. The replicative polymerase pol α synthesises short strands of RNA that prime DNA replication of the leading strand by the processivity factor PCNA bound pol ε and the lagging strand by pol δ [13]. Polymerase ε has been shown to be essential for helicase activation and remains associated with the CMG complex [14]. Interestingly, there is recent evidence that pol δ is required for the proper balance of the leading and the lagging strand synthesis coupled with the hypothesis that it may have played an important role in DNA synthesis of the leading strand when the cells exhibit replication stress [15]. Using purified *S.cerevisiae* replication proteins, it was discovered that the basic requirement for DNA replication initiation involves 16 replication factors alongside cyclin A-CDK2 and DDK phosphorylation [16]. It is to be noted that that study involved using replisome protein Mrc1 and Csm3/Tof1, to stabilise the activity of Mrc1 *in vitro*, to be able to achieve replication rates similar to those observed in vivo [16].

In normal cells, when an event leading to replication stress occurs, the DNA damage response is activated leading to the arrest of the cell cycle until DNA can be effectively repaired or the marking of cells for apoptosis. The marking of cells for apoptosis occurs to provide an effective way to prevent cells from progressing further into tumourigenesis. To protect the information encrypted within the DNA against damage and preserve genomic stability, eukaryotic cells have evolved the DNA structure checkpoint signalling pathways which are often referred to as the DNA-damage response (DDR) [17]. During replication stress, long stretches of ssDNA occur due to the uncoupling and dissociation of replicative polymerases and helicases [18]. This causes ssDNA to become coated with RPA to facilitate the recruitment of ataxia telangiectasia and Rad3-related protein (ATR) which phosphorylates various proteins involved in cell cycle arrest [5, 19]. The DNA damage response has been utilised by tumour cells to survive replicative stress by using the same proteins in ATR and CHK1. This occurs as the activation of ATR and CHK1 prevents the exposure of longer length ssDNA and thus prevents the collapse of replication forks. It has been confirmed that the activity of both ATR and CHK1 is required to stabilise DNA with RPA, thereby, preventing the collapse of replication fork [20, 21].

During a typical process of chromosome replication, replication forks can pause briefly when faced with sites where non-nucleosomal proteins are bound tightly. Any failure to stabilise these stalled replication forks can result in their collapse and thus, the monitoring of ongoing forks is essential for maintaining the integrity of the genome. Stalled replication forks need to be resolved in a swift manner by the cellular surveillance systems to be able to prevent the forks from collapsing and eventually complete the replication process [22, 23]. In *S. pombe*, the DNA structure checkpoints can be categorised into the DNA replication checkpoint and the DNA damage checkpoint. The major difference is that the DNA replication checkpoint prevents DNA damage in response to replication stress during S-phase, whilst the DNA damage checkpoint detects and resolves DNA damage before entry into mitosis [24]. An essential checkpoint response involves the arrest of the cell cycle by temporarily reducing CDK levels using CDK inhibitors and Cdc25 activity [24, 25]. In *S. pombe*, the DNA replication checkpoint depends primarily on the Rad3/Cds1 cascade pathway. This occurs as the replisome unwinds DNA downstream of the stalled replication fork to expose ssDNA structures to allow the loading and activation of the phosphatidylinositol 3-kinase-related protein kinase Rad3 in complex with Rad26 to the stalled replication fork areas [26–28]. Another independent reaction also occurs as the heterotrimeric Rad9-Rad1-Hus1 clamp is loaded using the Rfc2-5-Rad17-like loading complex to be able to fully activate Rad3 and Cds1 [29]. Cds1 is activated by a phosphorylation network composed of one basal and three parallel phosphorylation steps mediated by Rad3, Mrc1 and Rad9 [30, 31]. Another response is the DNA replication checkpoint transcriptional response, which includes the maintenance of the MluI cell cycle box (MCB) binding factor (MBF)-dependent G1/S transcriptional network [32]. This eventually causes the activation of MBF-dependent transcription which is essential for the survival of cells in response to replicative stress [33].

It was identified that the Rad4 is conserved across eukaryotes and that it is an essential gene as multicellular organism cells exhibit lethality when its function is disrupted [34, 35]. Expression of the *cut* mutant phenotype requires the viable function of several M phase regulators. This occurs due to the role of Rad4 in checkpoint control pathway and that role was characterised and developed by previous studies [36–41]. Fission yeast cut mutations disrupt coordination between M phase and cytokinesis, and cell division takes place in the absence of normal nuclear division [42]. There are approximately 20 cut genes known; however, DNA synthesis is not inhibited in any *cut* mutants except *cut5.*^**39,42**^ The *rad4-116* allele mimics conditions of replication stress in the absence of checkpoint function which makes it an attractive allele to utilise to study the genetic interactions of *rad4* [43].

During the S phase, nucleosomes are removed before the arrival of the replication machinery on the replication fork and then, nuclesomes are reassembled onto the newly synthesised DNA strand [44]. The assembly and removal of the nuclesomes also occur during transcription, recombination and repair and this is all mediated by the histone chaperone protein family [44]. Hip1 is one of the members of an evolutionarily conserved family of histone chaperones that can act independently from replication [45]. Furthermore, it has been reported that the *S.cerevisiae* HIR complex interacts with nucleosomes and prevents the remodelling activity of the SWI/SNF complex [46].

It has been shown that some factors are essential for DNA replication accuracy, but not DNA synthesis, as they travel with the moving replication fork. Two of those factors are Swi1 and Swi3 which are components of the fork stabilisation complex (FPC) [47–49]. The absence of Swi1 or Swi3 in cells leads to the accumulation of abnormal fork structures as observed with increased Rad52 DNA repair foci formation and recombination structures during the S phase [50]. The Swi1-Swi3 complex directly interacts with the DNA structure and recruits Mrc1 to the replication fork [14, 51]. The FPC also coordinates leading strand and lagging strand DNA synthesis as well as coordinating the DNA polymerase and helicase activity coupling at the replication forks [48, 49, 52]. Activation of the DNA damage response kinases in response to fork stalling agents requires the mediator protein Mrc1 [53–56] as it is present at replication forks even in the absence of DNA damage and is required for normal rates of progression of replication forks [57–60]. Swi3 is able to promote the efficient restart of stalled replication forks in a checkpoint-dependent manner as well as restoring broken replication forks in a checkpoint-independent manner [61]. Mrc1 is a conserved replication fork factor which is required for the stabilisation of stalled replication forks. In a manner independent of Cdc45 and Hsk1 kinase, it binds to early-firing origins [62]. Furthermore, Mrc1 also functions as a replication checkpoint mediator that allows Rad3-Rad26 to activate the effector kinase Cds1 and also interacts with Swi1-Swi3 on stabilisation of stalled replication forks [63, 64]. In the presence of fork stalling agents, Mrc1 is required to prevent continued replisome progression in the absence of DNA synthesis [65].

The aim of the study was to identify the network of genetic interactions of *rad4* in *S.pombe* that affect its DNA replication and checkpoint functions leading to synthetically lethal phenotypes. These interactions will be explored with both *rad4* and *rad4-116*, the temperature-sensitive *rad4* mutation, using molecular techniques. Once the strains are characterised, the *rad4* genetic interactions were mapped.

## Methods

### Yeast strains

Table 1 showing the *S. pombe* strains utilised with the corresponding genotype. The strains obtained from the Bioneer library(Bioneer Corporation, Daejeon, South Korea) are described in this paper [66].

**Table 1:** The fission yeast strains utilised in this study

Strain number	Genotype	Source
SPSC 120	*rad4-116 ura4-D18,leu1-32,ade6-M216 h+*	Collection
SPSC 573	*rad4-GFP::kanMX cdc25-22 ura3-D18 leu1-32 ade6 h-*	Collection
SPSC 1003	*ade6-M210 leu1-32 ura4-D18 mat1_m-cyhS mt0 rpl42::cyhR(sP56Q) rad4-116::NatMX h-*	Collection
SPSC 1005	*ura4-D18, leu1-32, ade6-704 h−*	Collection
SPSC 1006	*ade6-704 ura4-D18 leu1-32 h+*	Collection
SPSC 1017	*swi3Δ::KanMX h+ ade6-M216 ura4-D18 leu1-32 h+*	[66]
SPSC 1018	*mrc1Δ::KanMX ade6-M216 ura4-D18 leu1-32 h+*	[66]
SPSC 1026	*ade6-M216 leu1-32 ura4-D18 hip1Δ::KanMX h+*	Collection
SPSC 1029	*rad4::NatMX ura4-D18 h-*	Collection
SPSC 1047	*swi3Δ::KanMX ura4D-18 leu1-32 ade6- h-*	This study
SPSC 1048	*mrc1Δ::KanMX ura4D-18 leu1-32 ade6- h-*	This study
SPSC 1049	*hip1Δ::KanMX ura4-D18 leu1-32 h-*	This study
SPSC 1051	*swi1Δ::KanMX h+ ade6-M216 ura4-D18 leu1-32 h+*	[66]
SPSC 1056	*ade6-704 ura4-D18 leu1-32 rad52-GFP::KanMX6 h-*	Collection
SPSC 1063	*ade6-704 ura4-D18 leu1-32 rad52-GFP::KanMX6 mrc1::KanMX*	This study
SPSC 1064	*ade6-704 ura4-D18 leu1-32 rad52-GFP::KanMX6 swi3::KanMX*	This study
SPSC 1065	*ade6-704 ura4-D18 leu1-32 rad52-GFP::KanMX6 hip1::KanMX*	This study
SPSC 1073	*ade6-(704 or 210) ura4-D18 leu1-32 rad52-GFP::KanMX6 mat1_m-cyhS, smt0; rpl42::cyhR (sP56Q) rad4-116::NatMX h-*	This study
SPSC 1095	*ura4-D18, leu1-32, ade6-704 leu1::hip1::SV5(x3)::ura4+ h−*	This study
SPSC 1096	*ura4-D18, leu1-32, ade6-704 leu1::swi1::SV5(x3)::ura4+h−*	This study
SPSC 1097	*ura4-D18, leu1-32, ade6-704 leu1::swi3::SV5(x3)::ura4+h−*	This study
SPSC 1098	*ura4-D18, leu1-32, leu1::swi1::SV5(x3)::ura4+swi1Δ::KanMX h−*	This study
SPSC 1099	*ura4-D18, leu1-32, leu1::swi1::SV5(x3)::ura4+swi1Δ::KanMX rad4-116 h+*	This study
SPSC 1100	*ura4-D18, leu1-32, leu1::hip1::SV5(x3)::ura4+ hip1Δ::KanMX h−*	This study
SPSC 1101	*ura4-D18, leu1-32, leu1::swi3::SV5(x3)::ura4+swi3Δ::KanMX h−*	This study
SPSC 1102	*leu1::swi3::SV5(x3)::ura4+ura4-D18, ade6-704 swi3Δ::KanMX rad4-116 h−*	This study
SPSC 1103	*leu1::hip1::SV5(x3)::ura4+ura4-D18, ade6-704 hip1Δ::KanMX rad4-116 h-*	This study
SPSC 1104	*leu1::swi3::SV5(x3)::ura4+ura4-D18, ade6-704 swi3Δ::KanMX rad4-116 h+*	This study
SPSC 1105	*leu1::hip1::SV5(x3)::ura4+ura4-D18, ade6-704 hip1Δ::KanMX rad4-116 h+*	This study
SPSC 1106	*rad52-GFP::KanMX6 leu1::swi1::SV5(x3)::ura4+ura4-D18, ade6-704 swi1Δ::KanMX rad4-116::NatMX*	This study
SPSC 1108	*rad52-GFP::KanMX6 leu1::swi3::SV5(x3)::ura4+ura4-D18, ade6-704 swi3Δ::KanMX rad4-116::NatMX*	This study
SPSC 1110	*rad52-GFP::KanMX6 swi1Δ::KanMX ura4-D18 leu1-32*	This study
SPSC 1112	*rad52-GFP::KanMX6 leu1::hip1::SV5(x3)::ura4+ ura4-D18, ade6-704 hip1Δ::KanMX rad4-116::NatMX*	This study
SPSC 1114	*leu1::mrc1::SV5(x3)::ura4+ura4-D18, ade6-704 h−*	This study
SPSC 1115	*leu1::mrc1::SV5(x3)::ura4+ura4-D18, ade6-704 mrc1Δ::KanMX h−*	This study
SPSC 1116	*leu1::mrc1::SV5(x3)::ura4+ura4-D18, ade6-704 mrc1Δ::KanMX rad4-116*	This study

### Plasmids

Table 2 showing the plasmids utilised with the corresponding description. The plasmids were constructed to be used in the amplification of the desired gene prior to the yeast transformation process.

**Table 2:** The plasmids used in this study.

Plasmid number	Description
808	*pINTL mrc1-PkC*
809	*pINTL swi1-PkC*
810	*pINTL swi3-PkC*
811	*pINTL hip1-PkC*

### Spore microdissection

Cells were mated on ME agar by crossing h+ and h− strains and adding sterile water. The plates were incubated at 26 °C for 3–5 days for growth and sporulation to form zygotic asci, which were picked for tetrad dissection. After sporulation, a loop of crossed cells was streak on fresh YES or EMM plates to pick individual asci and place each spore in a grid matrix using the microdissection microscope Singer MSM System Series 400 (Singer Instruments, Somerset, UK). The four spores of the zygotic ascus were placed each at a unique position in a straight line using the micromanipulator. The plates were then incubated at 26 °C. After colony formation, the plates were replicated onto YES and more importantly, selective plates of YES with an antibiotic or EMM without specific supplements using filter paper (GE Healthcare Life Sciences, Little Chalfont, UK). When the spores grow after incubation at 26 °C, the plates are photographed using a ChemiDoc (Bio-Rad Laboratories Ltd., Watford, UK) for scoring and following genetic patterns. The desired genetic backgrounds for each colony was confirmed using PCR analysis. The viability of colonies on each plate was noted and total viable colonies were counted to calculate percentage viability and to exam the possibility of synthetic lethality by using the chi-square test.

### Statistics

The chi-square test was used to assess the possibility of synthetic lethality in experimental crosses. The viable cells or spores carried double drug-resistance markers, linked to genes, were counted as true observed value [O] while the expected value [E] was either calculated depending on the genetic segregation of each cross in most cases or in rare cases, as 25% of the total valid cells which include both the viable cells and the dead or unviable cells. Unviable cells are caused by either synthetic lethality or as errors in growth due to media content or micromanipulation errors, in these cases, the chi-square test eliminates the possibilities of anything but death from synthetic lethality. The general formula for the calculation of the chi-square value is as follows:

$$ {X}^2=\sum \frac{\left(\mathrm{Observed}\kern0.5em \mathrm{Value}\hbox{-} \mathrm{Expected}\kern0.5em \mathrm{Value}\right)2}{\left(\mathrm{Expected}\kern0.5em \mathrm{Value}\right)} $$

The probability associated with the value obtained from this test can be compared to a set of values from a *χ2* table for [*n*−1] degrees of freedom where [*n*] is the number of categories. For example, in the cases where the degree of freedom value is 1, if the *χ2* value is greater than 3.84, it can be deduced that the difference between the observed and expected value is statistically significant [*p* ≤ 0.05] and vice versa.

### Lithium acetate yeast transformation

*S. pombe* cells were transformed using the lithium acetate transformation method as described by Okazaki and coworkers [67].

### Hydroxyurea block and release

Cells were grown in YES to mid-log phase to the same OD as measured by a spectrophotometer (typically ~ 6 × 10^6^ cells/ml). Fresh 1 M hydroxyurea (Sigma-Aldrich Company Ltd., Gillingham, UK) was made up in YES and then added to the growing cultures to reach 10 mM final hydroxyurea concentration at a dilution factor of 1:100. At the 0 timepoint and after growth in hydroxyurea for 6 h, the cells were precipitated by spinning down in centrifuge at 4000 rpm. Then, the supernatant was discarded and the pellet washed twice with sterile water. The cell pellets were then used for the immunofluorescence protocol.

### Fluorescence microscopy and immunofluorescence

Cells were prepared for microscopy by fixing in 70% v/v ethanol. Welled microscope slides were used. The wells were filled with 30 g/100 ml agarose solution in sterile water. The agarose solution was boiled for 4 min then kept in a 70 °C water bath. The cells were then placed on the agarose-filled wells of the glass microscope slide. A solution of μg/ml 4′,6-diamidino-2-phenylindole (DAPI) and Vectashield (Vector Laboratories Ltd., Peterborough, UK) is then added to each well. DAPI binds strongly to DNA and is excited by ultraviolet light which then emits as a blue/violet colour. The coverslip was sealed with clear nail varnish and the cells visualised. Cells were observed using the Applied Precision Deltavision microscope and the images were deconvolved using the deconvolution software. The immunofluorescence technique is described in the paper by Hagan and Hyams [68].

### RNA extraction and reverse transcriptase real-time PCR

The RNA extraction and purification protocol was performed according to the manufacturer’s manual of the RNA extraction kit (MCR85102) (Epicentre, Chicago, USA). The Reverse transcriptase SensiFAST SYBR No-ROX kit (BIO-98005)(Bioline Reagents Limited, London, UK) reactions were performed according to the manufacturer’s instructions.

### Protein extraction, SDS-PAGE and western blotting

Cells were pelleted by centrifugation at 4000 rpm for 5 min. The cells were processed immediately after centrifugation or pellets were frozen at – 80 °C. The cells were then resuspended such that 100 μl of Y-PER™ Yeast Protein Extraction Reagent (78990) (Thermo Fisher Scientific Inc., Altrincham, UK ) were used to resuspend 50 mg of dry cell pellet. SDS PAGE gel electrophoresis was carried out using Bio-Rad Mini Protean II kits (Bio-Rad Laboratories Ltd., Watford, UK). The western blotting technique is based on the technique described by W. N. Burnette [69].

### The phenotypic characterisation of the V5-tagged strains

A V5-tagged version of the tested gene under transcriptional control of the nmt81 promoter was created using plasmids p808 to p811, as shown in Table 2, and integrated at the *leu1* locus following the methodology described by Fennessy and colleagues [70]. The V5-tagged base strain was constructed by transformation using SPSC 1005 as the transformed strain. The V5- tagged allele was tested using PCR and by performing a western blot of protein samples of an overnight culture of the new strain growing in EMM + uracil + leucine broth. By crossing and selecting *ura*^+^ G418-resistant colonies, the strain carrying V5-tagged version of the tested gene and the deleted copy at the original gene locus was constructed. This strain was then used in a cross with SPSC 120 to introduce the *rad4-116* allele by selecting *ura*^+^ G418 resistant temperature-sensitive colonies. The *rad52-GFP*-carrying strain was created by crossing. All the strains carrying the V5-tagged allele were tested using PCR before using them in the experimental procedures. The aim of this experiment was to be able to analyse the phenotypic profile of V5-tagged allele depletion for each strain in the presence of *rad4-116* with control strains carrying V5-tagged allele as well as a control strain carrying *rad4*^*+*^ and a control strain carrying *rad4-116*. The experiment is performed at the permissive temperature, 26 °C, so that it can mimic the conditions of the restrictive temperature when paired with the targeted gene deletion (*hip1*, *swi1*, *swi3* or *mrc1*). All the strains were grown into cultures up to 0.8OD overnight at 26 °C in EMM+uracil+leucine (no thiamine) then inoculated into a total volume of 200ml EMM + uracil + leucine + 50 μg/ml thiamine. The cultures were then incubated at 26 °C in a shaking incubator. Samples for RNA and protein extraction were collected at 0,4, 8, 12 and 24 h till the cultures for both experimental strains reached 1.00 OD from 0.2 OD. At each time point, samples for immunofluorescence were taken by fixing in formaldehyde. The viability of each strain was tested at each time point by plating 100 cells on EMM + uracil + leucine + 50 μg/ml thiamine agar plates in triplicates. The pellets for protein and RNA extraction were snap-frozen in liquid nitrogen and stored at – 80 °C. SPSC 120 (*rad4-116*) and SPSC 1005 (*rad4*^*+*^) were used as controls, with SPSC 120 being sampled every time the experiment was repeated. The experiment was repeated four times under identical conditions and the results show the averages of the repeats. The term used for the start of the experimental procedure is thiamine-induced induction of the repression of the selected gene and shortened to “thiamine induction”.

### The depletion of the V5-tagged allele expression by RT-QPCR

RT-QPCR was used to be able to assess the depletion of the transcript of the V5-tagged allele in the strains carrying V5-tagged allele. RNA was extracted from each strain at each time point and then purified from DNA and proteins. 50 ng of RNA of each sample was loaded per well in triplicates to able to obtain a signal for the V5 sequence. A control triplicate was loaded for the same sample to able to assess the transcription level of actin (*act1*). For each sample, negative control using no reverse transcriptase, negative control using no RNA sample and a negative control using no primers were loaded onto the same plate as the experimental wells. Each experiment was carried out in triplicate using separate samplings. Using the average readings for the V5 signal and the actin, the adjusted relative quantity of V5 transcript in each sample was calculated. The formula for the adjusted relative quantity value:

$$ \mathrm{Adjusted}\ \mathrm{relative}\ \mathrm{quantity}=\left(\mathrm{Relative}\ \mathrm{Quantity}\ \mathrm{Value}\ \mathrm{for}\ \mathrm{Sample}\right)-\left(\mathrm{Relative}\ \mathrm{Quantity}\ \mathrm{Value}\ \mathrm{for}\ \mathrm{Sample}\ \mathrm{without}\ \mathrm{Reverse}\ \mathrm{Transcriptase}\right)\times \left(\frac{\mathrm{The}\ \mathrm{Highest}\ \mathrm{Relative}\ \mathrm{Quantity}\ \mathrm{Value}\ \mathrm{for}\ \mathrm{act}1\ \mathrm{of}\ \mathrm{the}\ \mathrm{same}\ \mathrm{set}\left(\mathrm{strain}\right)}{\mathrm{Relative}\ \mathrm{Quantity}\ \mathrm{Value}\ \mathrm{for}\ \mathrm{act}1\ \mathrm{of}\ \mathrm{the}\ \mathrm{same}\ \mathrm{sample}\ }\right) $$

### The depletion of the V5-tagged allele expression by western blotting

The protein was extracted from each strain at each time point and then purified using the Y-PER™ Yeast Protein Extraction Reagent (78990) (Thermo Fisher Scientific Inc., Altrincham, UK). Proteins separated by SDS PAGE were then transferred onto Amersham Hybond™-PVDF membranes using a transfer cell (Bio-Rad Laboratories Ltd., Watford, UK) and then probed using the primary antibodies. Each PVDF membrane was separated based on the expected signal size and each separate section was probed with its corresponding antibody. After secondary antibody probing, each PVDF membrane was wrapped in saran wrap and the chemiluminescent signal was detected and imaged using the Bio-Rad Chemidoc XRS+ Imaging System (Bio-Rad Laboratories Ltd., Watford, UK) or using an X-ray film. For each strain, the western blotting was repeated at least three times using separate samplings.

## Results

### Synthetic lethality on agar

The aim of the experiment was to investigate the interactions between *rad4* and *hip1* which is implicated in the maintenance of chromatin structure and remodelling. The strains selected had either the *rad4-116* marked with NAT resistance and the *hip1Δ* marked with G418 resistance. This was achieved by mating the strains, after changing the mating type where needed, on EMM-nitrogen agar plates then micromanipulating and dissecting the tetrads, followed by replicating the plates on the appropriate selective media. Tetrad dissection provided clear and separated colonies on the plates, thus, facilitating the assessment of viability and avoiding contaminants forming colony-like growth. The tetrads could segregate as parental di-type, non-parental di-type or as a tetra-type depending on the combination of alleles inherited by each spore. The growth of colonies on YES indicates that the interaction between the genes present in the original spore was not synthetically lethal whereas the spores that were not able to form colonies indicate that a synthetically lethal interaction occurred in the double mutant. The original YES was replicated onto a fresh YES plate as well as YES + NAT, YES + G418 and YES + NAT + G418. The colonies were counted and the growth patterns were observed and noted for each plate and for each cross. The *χ2* was then calculated based on the expected number of viable spores.

**Fig. 1:**
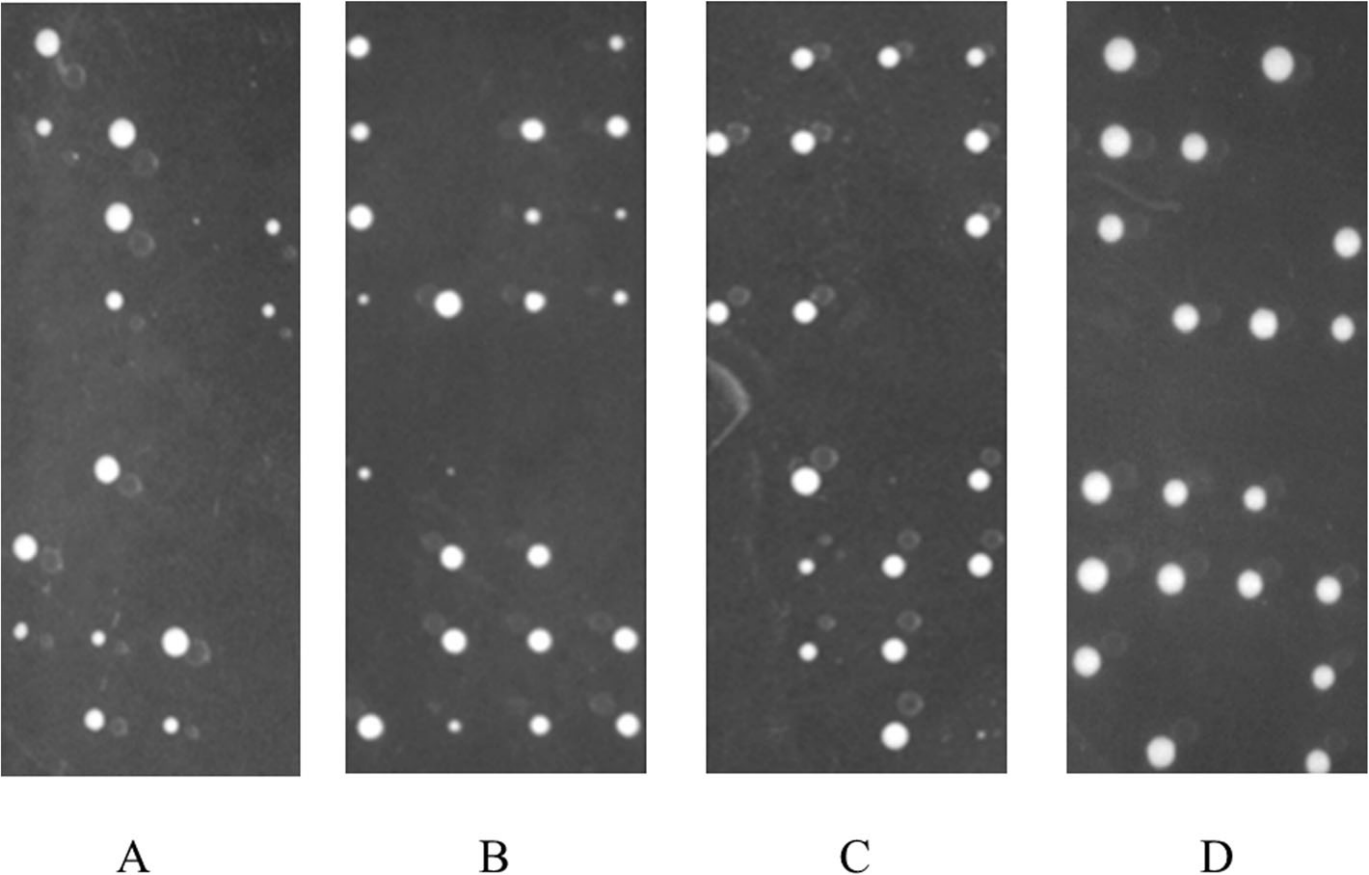
The growth of individual spores to colonies of the *rad4-116* strain cross with single deletion strains. **a** The growth of individual spores to colonies of the *rad4-116* strain cross with *hip1Δ* strain. **b** The growth of individual spores to colonies of the *rad4-116* strain cross with *swi1Δ* strain. **c** The growth of individual spores to colonies of the *rad4-116* strain cross with *swi3Δ* strain. **d** The growth of individual spores to colonies of the *rad4-116* strain cross with *mrc1Δ* strain. The figure shows 4 columns of the YES plates of the crosses of the *rad4-116* strain with a *swi1Δ* strain. The cross shown was a set of spores from tetrads that were inoculated into single spores with a line separating each individual spore set from a single tetrad. The number of spores from each YES plate was counted and recorded

After the colonies from the crosses were noted and counted from all the plates, as seen in Fig. 1, the chi-square test was performed as shown in Table 3. As this value was calculated using the expected value based on the segregated spores not based on the expected segregation pattern, the values represent a focused measure on whether the double mutants were synthetically lethal or not. These values were all greater than 3.84, which was the threshold value at *p* ≤ 0.05 with a degree of freedom of 1. This indicated that the deletion of *hip1*, *swi1*, *swi3* and *mrc1*, separately were synthetically lethal when combined with *rad4-116* separately, and that any lethality or observed lack of growth would not be due to chance or random occurrence.

**Table 3:** The crosses between the *rad4-116* strain and single deletion strains

Cross	*hip1Δ* × *rad4-116*	*swi1Δ* × *rad4-116*	*swi3Δ* × *rad4-116*	*mrc1Δ* × *rad4-116*
Wild-type	32	32	45	35
G418^R^	35	42	51	46
NAT^R^	30	32	46	41
G418^R^/NAT^R^	4	14	1	12
Viability	82	125	143	132
Total valid spores	144	192	212	180
*χ*^*2*^ value	27.7	23.4	22.5	12.8

Table 3 shows the crosses between the *rad4-116* strain and single deletion strains. The *rad4-116* was tagged with NAT resistance while the *mrc1* deletion was marked with G418 (Kanamycin) resistance. The crosses were carried out on EMM-nitrogen agar, then checked after 2 days for sporulation. The tetrads from sporulation were separated using the Singer Micromanipulator onto YES agar plates. After 4 days, the plates were then copied onto fresh YES, as shown in Fig. 1, YES+G418 and YES+NAT plates separately. After another 3 days, the colonies on each plate were counted and recorded as seen in the table. Viability was measured by counting the observed colonies on the YES agar plates. Therefore, those colonies were able to be copied and re-grown for analysis. The *χ*^*2*^ value for each cross was calculated and tabulated at *p* ≤ 0.05 with a degree of freedom of 1.

### Hydroxyurea block and release

Cells were grown in liquid media then arrested by the addition of hydroxyurea which blocks ribonucleotide reductase leading to the depletion of the ribonucleotides, halting DNA replication. Strains carrying separate mutations of *rad4-116* or deletions of *hip1*, *swi1*, *swi3* and *mrc1* were crossed with strains expressing Rad52-GFP to create the experimental strains for this procedure. The use of Rad52-GFP fluorescent foci represented an effective method to assess the accumulation of stalled or collapsed fork events during replication as shown in previous studies [50]. Then, after 6 h of arrest with hydroxyurea, the cells were released by the depletion of hydroxyurea from the media, washing the cells, then resuspending in rich YES media. Samples were collected every 30 min for 210 min to be analysed for fluorescence microscopy and testing viability. The timepoint 0 represents the first time point after resuspension of the culture in YES media. All of the procedures including culturing, washing, resuspension and sampling were carried out at 26 °C.

**Fig. 2:**
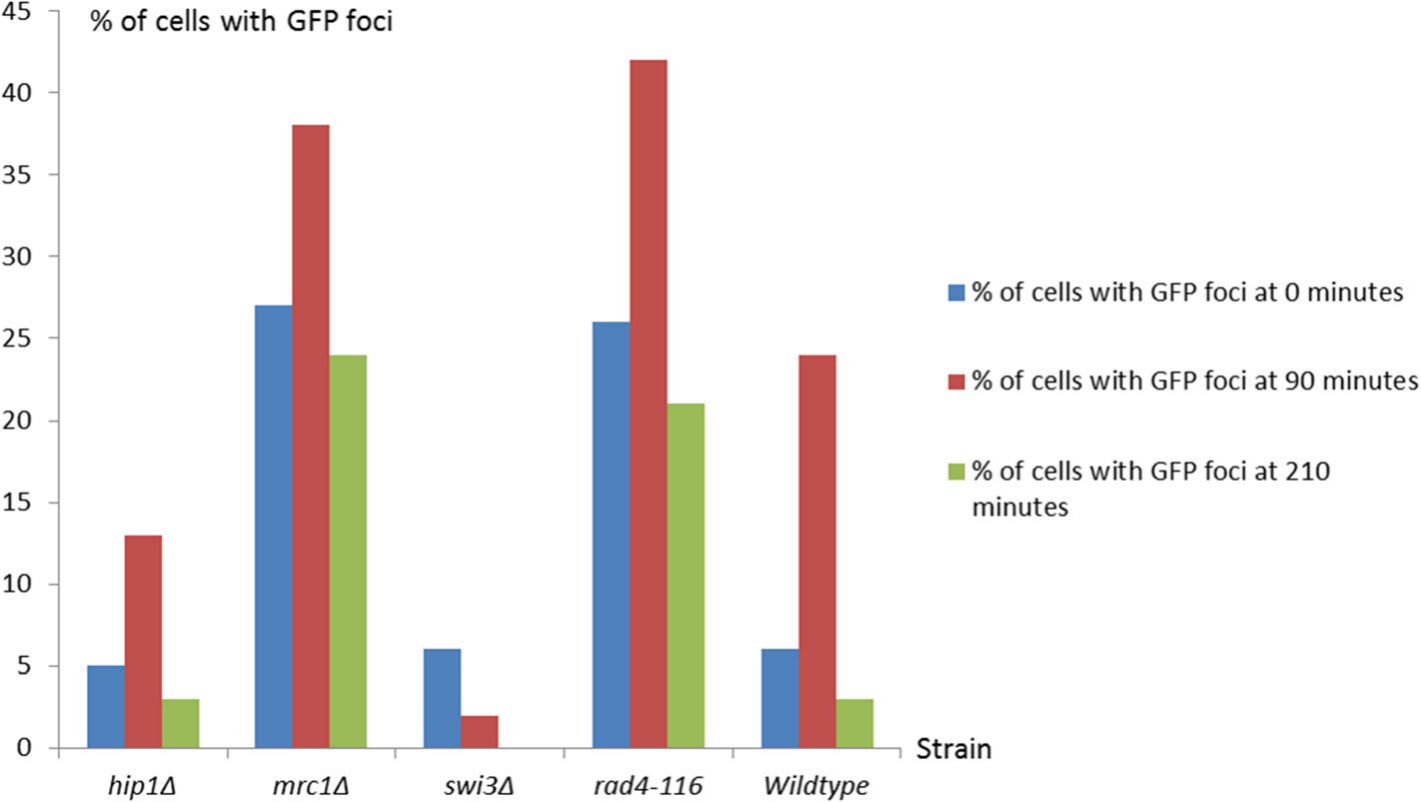
A comparison of the Rad52-GFP profiles of the *hip1Δ*, mrc1Δ, *rad4-116*, *rad4*^*+*^ and *hip1Δ* strains. The figure shows the bar chart graph representing the comparison between the percentage of cells with Rad52-GFP foci across the strains at the start of the experiment, at 90 min after the start and at the end of the experiment at 210 min

The cell nuclei were assessed by observing and calculating the number and of nuclei that carry GFP fluorescent foci spanning the 210 min compared to the starting time point (0) of the release. The number of nuclei carrying GFP foci was divided over the total number of nuclei to provide the percentage observed in the graphical representation in Fig. 2. It is important to note that none of the strains with Rad52-GFP showed a decrease in viability across the 210-min timecourse.

### The phenotypic characterisation of the V5-tagged strains

### The viability of the V5-tagged strains across 24 h

**Fig. 3:**
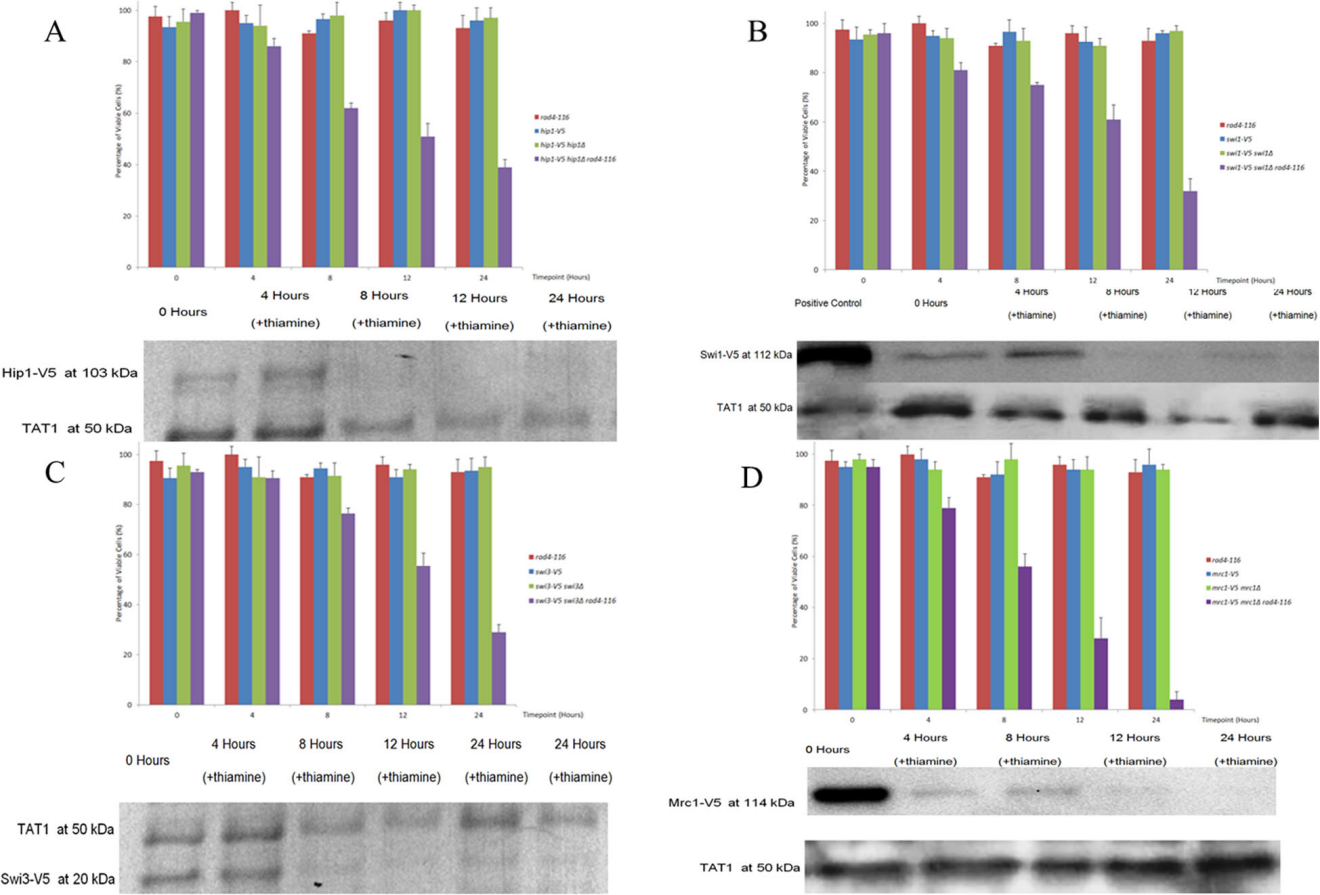
The graphical representation of the viability comparison of strains carrying the *V5* alleles against the control *rad4-116* strain across the 24-h timecourse after the addition of thiamine to the media. For the strains carrying both the *V5* alleles, deletions of the wildtype genes and the *rad4-116* gene, the western blots of the depletion of V5 tag are shown. **a** Among all the *hip1-V5* strains, only SPSC 1103 showed a gradual loss of viability dropping from 99% viability at 0 time point to 51% after 12 h, then to 39% viability at 24 h. All the other strains represented showed no significant loss of viability across the 24-h timecourse ranging from 100 to 91% viability. The western blot of SPSC 1103 shown represents the samples after being probed with anti-V5 (top signal) and anti-TAT1 (bottom signal) as control. The 0- and 4-h samples expressed the V5 tag while there was no visible signal for the 8-, 12- or 24-h samples. The TAT1 signal showed no signs of depletion across all 5 samples. **b** Among all the *swi1-V5* strains, only SPSC 1099 showed a gradual loss of viability dropping from 96% viability at 0 time point to 61% after 12 h, then to 32% viability at 24 h. All the other strains represented showed no significant loss of viability across the 24-h timecourse ranging from 100 to 91% viability. The western blot of SPSC 1099 shown represents the samples after being probed with anti-V5 (top signal) and anti-TAT1 (bottom signal) as control. The 0, 4 and 8-h samples expressed the V5 tag while there was no visible signal for the 12- or 24-h samples. An additional positive control SPSC 1099 sample is shown on the right where no thiamine is added. **c** Among all the *swi3-V5* strains, only SPSC 1102 showed a gradual loss of viability dropping from 93% viability at 0 time point to 56% after 12 h, then to 29% viability at 24 h. All the other strains represented showed no significant loss of viability across the 24-h timecourse ranging from 100 to 91% viability. The western blot of SPSC 1102 shown represents the samples after being probed with anti-TAT1 (top signal) and anti-V5 (bottom signal) as control. The 0- and 4-h samples expressed the V5 tag while there was no visible signal for the 8-, 12- or 24-h samples. An additional sample at 24 h was taken to underline the complete depletion of the V5 tag. **d** Among all the *mrc1-V5* strains, only SPSC 1116 showed a gradual loss of viability dropping from 95% viability at 0 time point to 28% after 12 h, then to 4% viability at 24 h. All the other strains represented showed no significant loss of viability across the 24-h timecourse ranging from 100 to 91% viability. The western blot of SPSC 1116 shown represents the samples after being probed with anti-V5 (top signal) and anti-TAT1 (bottom signal) as control. The 0-h sample strongly expressed the V5 tag while the 4- and 8-h samples expressed very weak V5 signals, but there were no visible signals for the 12- or 24-h samples

With regard to the depletion phenotypes of strains with *rad4-116* backgrounds, the viability profiles for Swi1 and Swi3 were most similar as observed in Fig. 3. All the strains carrying the gene deletion, the V5-tagged allele of the same gene and the *rad4-116* gene showed a gradual decrease in viability from time point 0 to time point 24 h. Strains with either the gene deletion (*hip1Δ*, *swi1Δ*, *swi3Δ*, *mrc1Δ*) and/or the V5-tagged allele (*hip1-V5*, *swi1-V5*, *swi3-V5*, *mrc1-V5*) and *rad4*^*+*^ showed no decrease in viability from timepoint 0 across the 24 h timecourse after thiamine addition as observed in Fig. 3. Figure 3 also contained a *rad4-116* control with no gene mutations or V5-tagged alleles that did not show any decrease in viability and was repeated every time a strain was sampled. The strain that showed the highest level of viability loss was the *mrc1Δ mrc1-V5 rad4-116* dropping from 95% at time point 0 to 4% after 24 h for a total loss of 91% as observed in Fig. 3. In one repetition of *mrc1Δ mrc1-V5 rad4-116* sampling, the three EMM agar plates showed no colonies growing with the expectancy of 100 viable colonies of each in case of 100% viability. In terms of V5 RNA and protein expression, all the strains with V5-tagged genes (*hip1-V5*, *swi1-V5*, *swi3-V5*, *mrc1-V5*) showed a gradual decrease across the 24 h after thiamine addition. Generally, using western blotting, the V5 signal is abolished at the 8- or 12-h timepoint with a strong signal observed at the initial time point 0 and a weaker signal observed at time point 4 h with no change observed in the α-Tubulin control protein expression. The decrease and eventual abolishment of the detectable V5-tagged protein signal is consistent with the decrease in V5 transcription of each gene. In addition, in the strains carrying the gene deletion, the V5-tagged allele of the same gene and the *rad4-116* gene, the depletion of V5 RNA (as seen in Supplementary Data Figure S1) and protein signals were consistent with the development of cellular phenotypes in terms of cell length, nuclear integrity and size, typically present at 12 h and more pronounced at 24 h.

In the experiment with *hip1Δ hip1-V5 rad4-116* strain SPSC 1103, two distinct phenotypes were observed at time points 12 and 24 h. The more common phenotype being longer of more than 10 μm in length and the less common phenotype being a shorter cell of less than 7 μm in length as observed in Fig. 5. Both phenotypes show irregular nuclear size and shape with some nuclei being elongated and others fragmented or compacted.

**Fig. 4:**
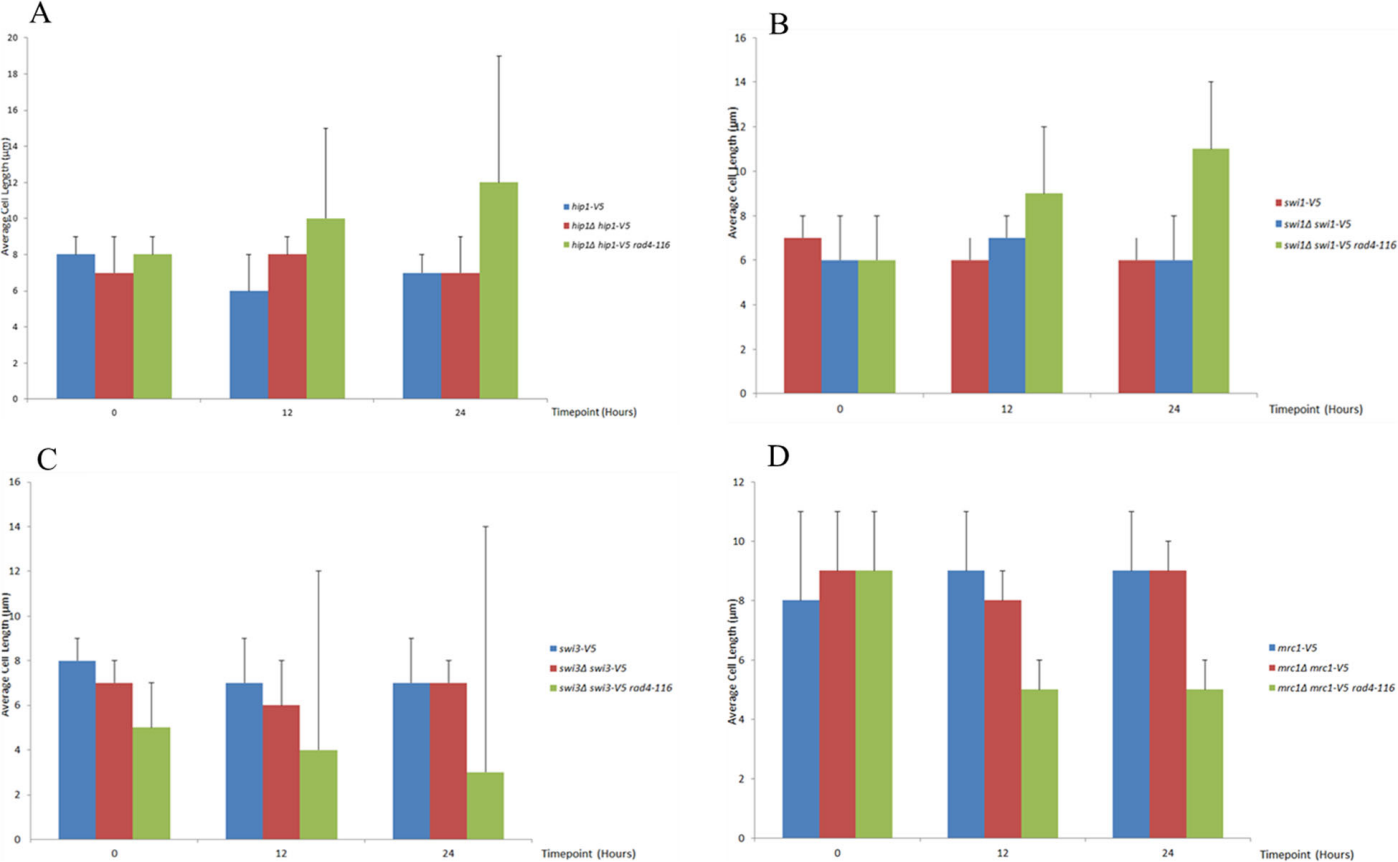
The average cell length of the strains carrying the *V5* alleles. **a** The graphical representation of the comparison of average cell length between the base *hip1-V5* strain, the *hip1-V5 hip1Δ* strain and the *hip1-V5 hip1Δ rad4-116* strain. **b** The graphical representation of the comparison of average cell length between the base *swi1-V5* strain, the *swi1-V5 swi1Δ* strain and the *swi1-V5 swi1Δ rad4-116* strain. **c** The graphical representation of the comparison of average cell length between the base *swi3-V5* strain, the *swi3-V5 swi3Δ* strain and the *swi3-V5 swi3Δ rad4-116* strain. **d** The graphical representation of the comparison of average cell length between the base *mrc1-V5* strain, the *mrc1-V5 mrc1Δ* strain and the *mrc1-V5 mrc1Δ rad4-116* strain

In the *swi1Δ swi1-V5 rad4-116* strain SPSC 1099, a single phenotype was observed at time points 12 and 24 h as seen in Fig. 6. A significant increase occurs from 6 μm to 9 μm at 12 h before reaching 11 μm at 24 h after the addition of thiamine as shown graphically in Fig. 4. The cells also show multiple cellular polarisation with a common phenotype being 3 cytoplasmic ends. The increase in cell length was accompanied by a fragmentation of nuclei and the emergence of compact nuclei and occasionally cells showing no DAPI staining indicating uneven nuclear division.

**Fig. 5:**
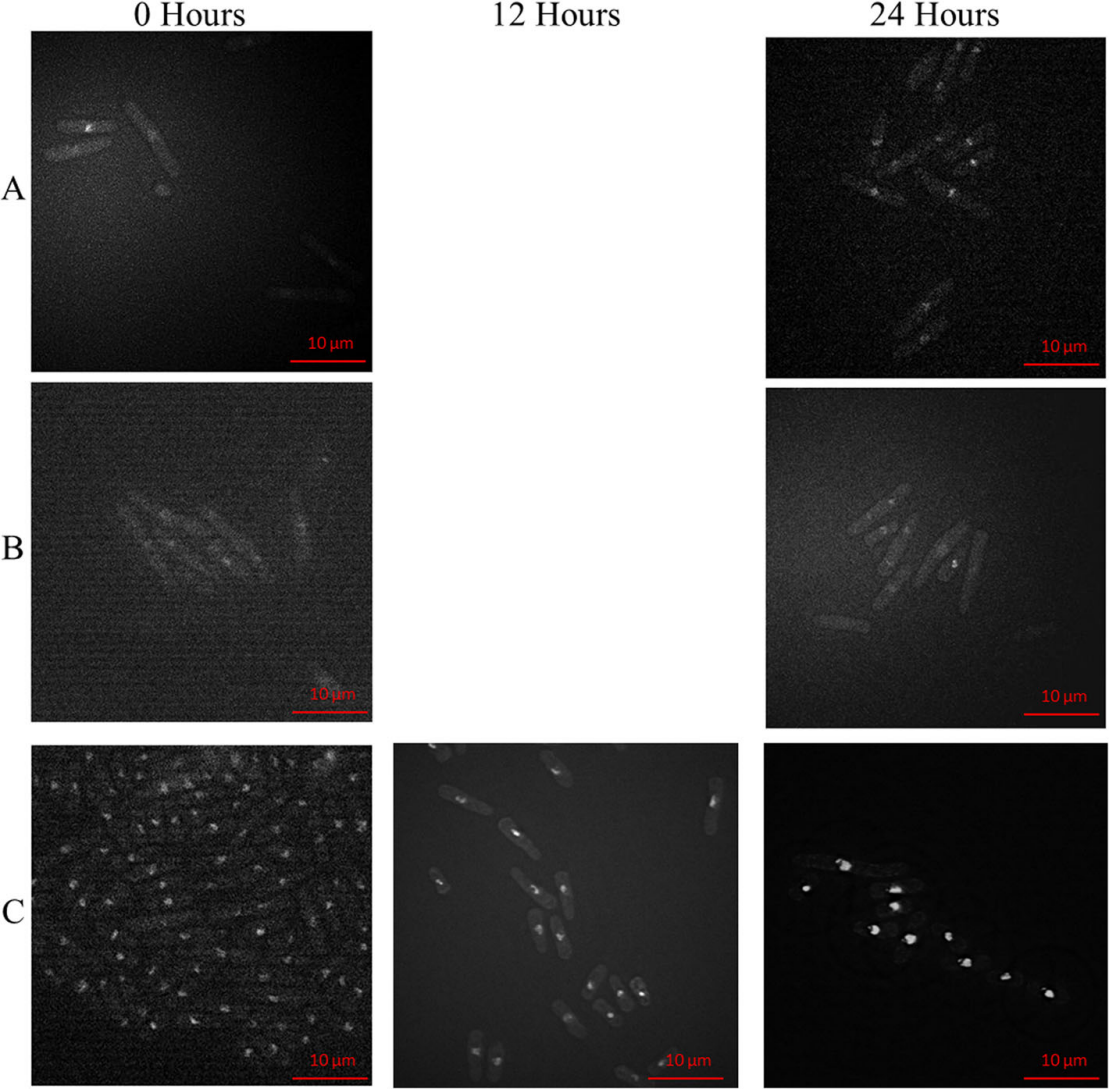
The cell morphology of the strains carrying the *hip1-V5* allele across 24 h after thiamine induction. The figure shows the cells of the **a***hip1-V5* strain SPSC 1095, **b***hip1Δ hip1-V5* strain SPSC 1100 and **c***hip1Δ hip1-V5 rad4-116* strain SPSC 1103 at × 60 magnification stained with DAPI to show the nuclei at the 0 h time point and the 24 h timepoint. An additional time point at 12 h is shown for the SPSC 1103 strain. For SPSC 1095 and SPSC 1100, there was no significant difference between the cells across those 2 time points in terms of cell length, cell shape and nuclear shape. For SPSC 1103, the cells showed two distinct phenotypes that develop from timepoint 0 to 12 to 24 h, for cells to increase or decrease in length. The nuclei of most of these cells showed a fragmentation that becomes more pronounced at the 24 h time point and some cells showed a compacted nucleus. The phenotypical effect of thiamine addition on the *hip1-V5* strains across 24 h

**Fig. 6:**
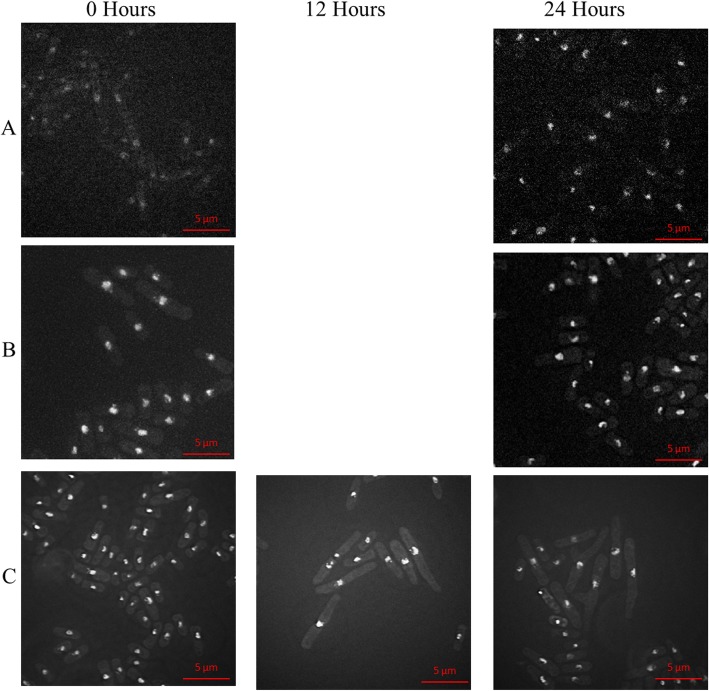
The cell morphology of the strains carrying the *swi1-V5* allele across 24 h after thiamine induction. The figure shows the cells of the (**a**) *swi1-V5* strain SPSC 1096, (**b**) *swi1Δ swi1-V5* strain SPSC 1098 and **c***swi1Δ swi1-V5 rad4-116* strain SPSC 1099 at × 100 magnification stained with DAPI to show the nuclei at the 0 h time point and the 24 h timepoint. An additional time point at 12 h is shown for the SPSC 1099 strain. For SPSC 1096 and SPSC 1098, there was no significant difference between the cells across those 2 time points in terms of cell length, cell shape and nuclear shape. For SPSC 1099, the cells showed a distinct phenotype, that developed from timepoint 0 to 12 to 24 h, for cells to increase in length. The nuclei of most of these cells showed a fragmentation that became more pronounced at the 24-h timepoint and some cells showed a compacted nucleus. Some mutant cells showed an abnormal cell shape with one nucleus and three ends to the cytoplasm. The phenotypical effect of thiamine addition on the swi1-V5 strains across 24 h

**Fig. 7:**
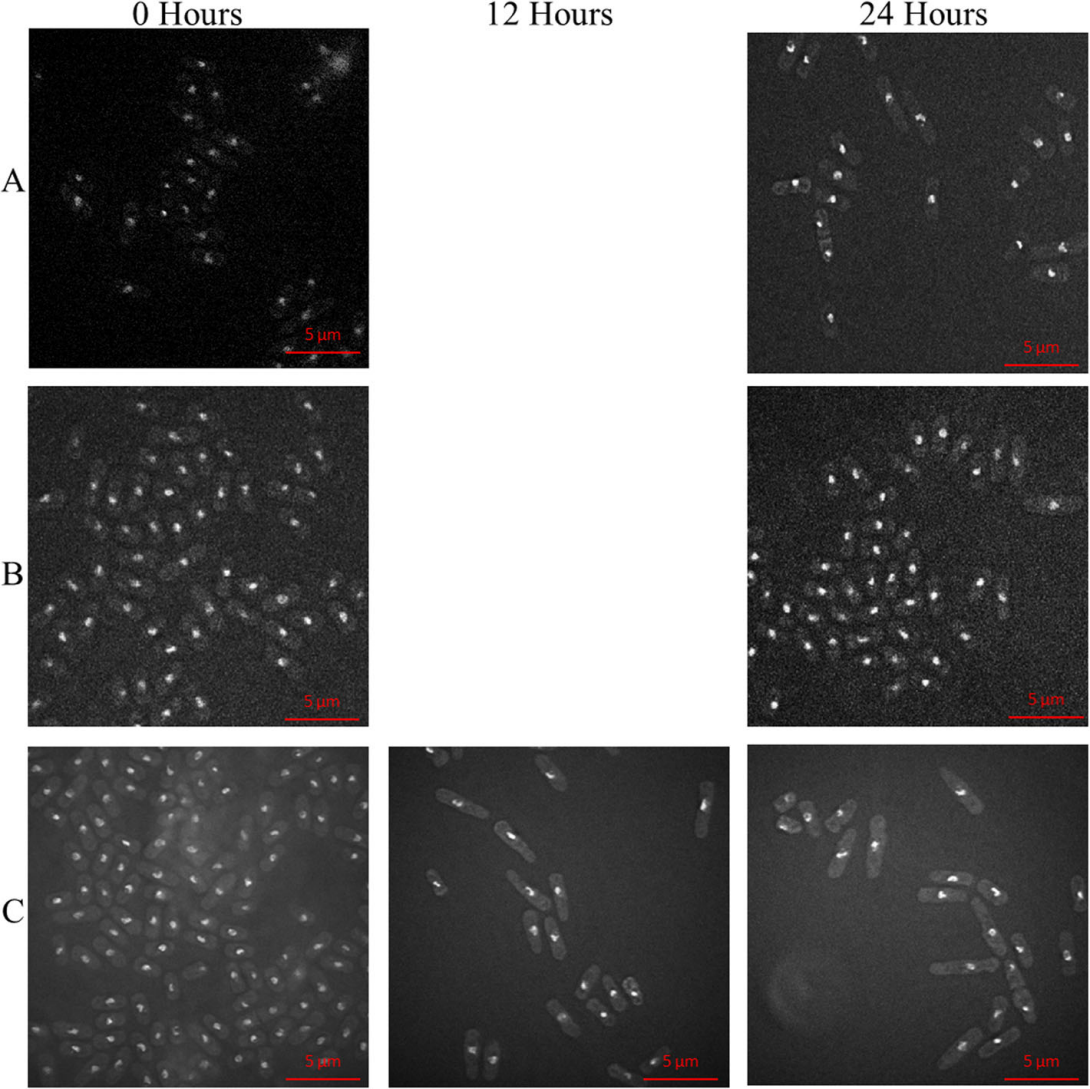
The cell morphology of the strains carrying the *swi3-V5* allele across 24 h after thiamine induction. The figure shows the cells of the (**a**) *swi3-V5* strain SPSC 1097 (**b**) *swi3Δ swi3-V5* strain SPSC 1101 and (**c**) *swi3Δ swi3-V5 rad4-116* strain SPSC 1102 at × 100 magnification stained with DAPI to show the nuclei at the 0 h time point and the 24 h timepoint. An additional time point at 12 h is shown for the SPSC 1102 strain. For SPSC 1097 and SPSC 1101, there was no significant difference between the cells across those 2 time points in terms of cell length, cell shape and nuclear shape. For SPSC 1102, the cells showed two distinct phenotypes, that developed from timepoint 0 to 12 to 24 h, for cells to increase or decrease in length. The nuclei of most of these cells showed irregular nuclear shapes that became more pronounced at the 24-h time point and some cells showed a compacted nucleus. The phenotypical effect of thiamine addition on the swi3-V5 strains across 24 h

With regard to the *swi3-V5 swi3Δ rad4-116* strain SPSC1102, two distinct phenotypes were observed at timepoints 12 and 24 h as seen in Fig. 7. The more common phenotype being shorter of less than 5 μm in length and the less common phenotype being a longer cell of more than 5 μm in length as observed in Fig. 7. Both phenotypes show irregular nuclear size and shape with some nuclei being elongated or compacted.

**Fig. 8:**
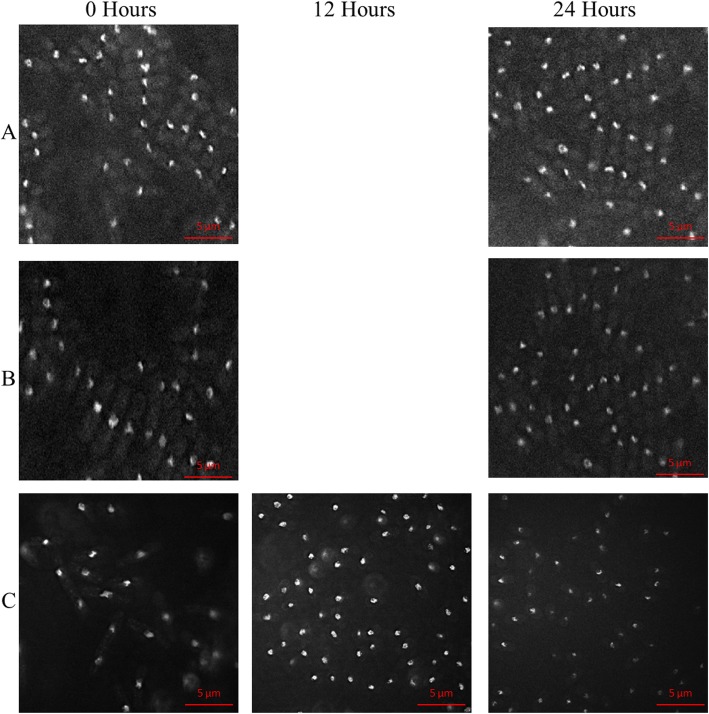
The cell morphology of the strains carrying the *mrc1-V5* allele across 24 h after thiamine induction. The figure shows the cells of the (**a**) *mrc1-V5* strain SPSC 1114 (**b**) *mrc1Δ mrc1-V5* strain SPSC 1115 and (**c**) *mrc1Δ mrc1-V5 rad4-116* strain SPSC 1116 at × 100 magnification stained with DAPI to show the nuclei at the 0-h and the 24-h timepoints. An additional time point at 12 h is shown for the SPSC 1116 strain. For SPSC 1114 and SPSC 1115, there was no significant difference between the cells across those 2 time points in terms of cell length, cell shape and nuclear shape. For SPSC 1116, the cells showed a distinct phenotype, that developed from timepoint 0 to 12 to 24 h, for cells to decrease in length. The nuclei of most of these cells showed a fragmentation that became more pronounced at the 24 h time point and some cells showed compacted nuclei. The phenotypical effect of thiamine addition on the mrc1-V5 strains across 24 h

As for the *mrc1-V5 mrc1Δ rad4-116* strain SPSC 1116*,* the cells show a distinct phenotype, that develops at timepoints 12 and 24 h as seen in Fig. 8. The initial average cell length at 0 is similar to the other two *mrc1-V5* strains, but then a significant decrease occurs from 9 μm to 5 μm at both 12 and 24 h after the addition of thiamine as observed in Fig. 4. The decrease in cell length was accompanied by fragmentation and compaction of nuclei with the shape of the nuclei.

### The effect of thiamine addition on the average cell length of the V5-tagged strains across 24 h

No significant change in average cell length occurred in both the base *hip1-V5* strain and the *hip1-V5 hip1Δ* strain as the value is between 6 and 8 μm between 0 and 24 h to finally reach 7 μm for both strains at 24 h after thiamine addition. As for the *hip1-V5 hip1Δ rad4-116* strain, the initial average cell length at 0 was similar to the other two strains at 8 μm. However, a gradual increase was then observed from 8 μm to 10 μm at 12 h then 12 μm at 24 h after the addition of thiamine. The cells of the *hip1-V5 hip1Δ rad4-116* strain contained two phenotypic cell sizes with the predominant one being an elongated cell of more than 13 μm in length and the other, less common, much shorter phenotype, being of 3 to 5 μm in length. The presence of high-value error bars indicated the presence of different phenotypes for the same strain.

No significant change in average cell length occurred in both the base *swi1-V5* strain and the *swi1-V5 swi1Δ* strain as the value is between 7 and 6 μm between the 0 and 24 h, eventually reaching 6 μm at 24 h after thiamine addition. As for the *swi1-V5 swi1Δ rad4-116* strain, the initial average cell length at 0 was similar to the other two strains at 6 μm, but then a significant increase occurred from 6 μm to 9 μm at 12 h before reaching 11 μm at 24 h after the addition of thiamine.

No significant change in average cell length occurred in both the base *swi3-V5* strain and the *swi3-V5 swi3Δ* strain as the value is between 6 and 8 μm between the 0 h and 24 h to finally reach 7 μm for both strains at 24 h after thiamine addition. As for the *swi3-V5 swi3Δ rad4-116* strain, the initial average cell length at 0 was lower than the other two strains at 5 μm. A gradual decrease was then observed from 5 μm to 4 μm at 12 h then 3 μm at 24 h after the addition of thiamine. The cells of the *swi3-V5 swi3Δ rad4-116* strain contained two phenotypic cell sizes with the predominant one being a short, almost spherical, cell of 2 to 3 μm in length and the other, less common phenotype, being of 9 to 12 μm in length. The presence of high-value error bars indicated the presence of different phenotypes for the same strain.

No significant change in average cell length occurred in both the base *mrc1-V5* strain and the *mrc1-V5 mrc1Δ* strain as the value is between 8 and 9 μm between the 0 h and 24 h after thiamine addition. As for the *mrc1-V5 mrc1Δ rad4-116* strain, the initial average cell length at 0 was similar to the other two strains, but then a significant decrease occurred from 9 μm to 5 μm at both 12 h and 24 h after the addition of thiamine.

## Discussion

The synthetic lethality data obtained by crossing *rad4-116* and *hip1Δ*, *swi1Δ*, *swi3Δ* and *mrc1Δ* separately provided confirmation of the preliminary SGAA screen. As observed in Table 1, all the crosses produced statistically significant synthetically lethal phenotypes. This further proves the synthetical interaction between *rad4-116* on one side and *hip1Δ*, *swi1Δ*, *swi3Δ* and *mrc1Δ* separately on the other side. The synthetic lethality profiles of *swi1Δ* and *swi3Δ* are nearly identical to each other in terms of the *χ*^*2*^ values being 23.4 and 22.5. The synthetic lethality profile of *hip1Δ* was also similar to *swi1Δ* and *swi3Δ* profiles with a *χ*^*2*^ value of 27.7. However, surprisingly, the synthetic lethality profile of *mrc1Δ* was not as similar to *hip1Δ*, *swi1Δ* and *swi3Δ* profiles in terms of *χ*^*2*^ value as the *χ*^*2*^ value was 12.8. Indeed, all the *χ*^*2*^ values were statistically significant showing lethality. The difference of synthetic lethality profiles is interesting given that Swi1, Swi3 and Mrc1 act to stabilise stalled replication forks [14, 51]. However, it should be noted that the Swi1-Swi3 complex recruits Mrc1 onto the replication fork [14].

In terms of the DNA replication stalled fork profiles, a similar pattern was observed. The *hip1Δ*, *swi1Δ* and *swi3Δ* DNA replication stalled fork recovery profiles are similar in terms of peak value of nuclei and recovery with Rad52-GFP foci as observed in Fig. 2. The DNA replication stalled fork recovery profiles of *swi1Δ* and *swi3Δ* were less similar than their synthetic lethality profiles with the *swi3Δ* strain showing a very low accumulation of Rad52-GFP foci even when compared to the *rad4*^*+*^ strain as observed in Fig. 2. Furthermore, a strong similarity was observed between the *rad4-116* and *mrc1Δ* DNA replication stalled fork profiles as observed in Fig. 2. The full profiles across the full 210-min timecourse showed the same pattern in terms of starting level of percentage of cells with Rad52-GFP foci, the increase up to a similar peak level, maintenance of a steady level of foci and final level observed at 210 min as illustrated in Fig. 2. These findings for *mrc1Δ* are consistent with those presented previously in *S.cerevisiae* [65]. The *rad4-116* and *mrc1Δ* strains are the only two strains that do not recover below the starting level of RAd52-GFP foci as observed in Fig. 2. The accumulation of Rad52-GFP in *swi1Δ* and *swi3Δ* strains were similar, albeit no reaching the same level of accumulation, as those noted in previous studies [50, 61, 71].

In the experiment with *hip1Δ hip1-V5 rad4-116* strain SPSC 1103, two distinct phenotypes were observed at timepoints 12 and 24 h. The more common phenotype being longer of more than 10 μm in length and the less common phenotype being a shorter cell of less than 7 μm in length as observed in Fig. 5. Both phenotypes show irregular nuclear size and shape with some nuclei being elongated and others fragmented or compacted. The fragmented nuclei, as observed in Figs. 4 and 5, were to be expected as the HIRA proteins, including Hip1, have been proven to be integral in histone modification and chromatin structure [46, 72].

In the *swi1Δ swi1-V5 rad4-116* strain SPSC 1099, the increase in cell length was accompanied by a fragmentation of nuclei and the emergence of compact nuclei and occasionally cells showing no DAPI staining indicating uneven nuclear division. This is consistent with the role of Swi1 (Tof1) in terms of function in paused fork resolution and for stable association of replisome components with sites of DNA synthesis in the presence of hydroxyurea [65, 73, 74, 75]. This may also indicate additional roles of Swi1 in terms of chromatin structure maintenance that may be affected by Rad4.

With regards to the *swi3-V5 swi3Δ rad4-116* strain SPSC1102, it should be noted that nuclear fragmentation was not observed unlike those observed in the *swi1Δ swi1-V5 rad4-116* and *hip1Δ hip1-V5 rad4-116* strains. The appearance of the shorter length phenotype not seen in the *swi1Δ swi1-V5 rad4-116* strain was interesting, indicating a function exclusive to Swi3 not present in Swi1. The difference in phenotypes between *swi1Δ swi1-V5 rad4-116* and *swi3-V5 swi3Δ rad4-116* was also interesting given that Swi1 and Swi3 act to stabilise stalled replication forks as the same complex [14, 51].

As for the *mrc1-V5 mrc1Δ rad4-116* strain SPSC 1116, the decrease in cell length was accompanied by fragmentation and compaction of nuclei with the shape of the nuclei similar to those seen in *swi1Δ swi1-V5 rad4-116* and *hip1Δ hip1-V5 rad4-116* strains. The phenotype in the *mrc1-V5 mrc1Δ rad4-116* strain was similar to the shorter phenotype of the *swi3-V5 swi3Δ rad4-116* strain in terms of cell length. This is consistent with the role of Mrc1 as a conserved replication fork factor and its interaction with Swi1-Swi3 complex [62, 64]. It is important to note the similarity in phenotype between the shorter phenotype of the *swi3-V5 swi3Δ rad4-116* strain and *mrc1-V5 mrc1Δ rad4-116* strain on the one hand and the similarity between longer phenotype of the *swi3-V5 swi3Δ rad4-116* and *hip1Δ hip1-V5 rad4-116* strain in terms of cell length. It should be noted that the *rad4-116* interaction with the gene deletions (*hip1Δ*, *swi1Δ*, *swi3Δ*, *mrc1Δ*) was most likely due to the development of stalled replication forks, with eventual fork collapse, and double strand breaks as the *rad4-116* allele mimics conditions of replication stress in the absence of checkpoint function [43]. However, that does not rule out additional interactions based on the other roles of Rad4 which led to the development of different and distinct phenotypes as the V5-tagged genes were deleted in the presence of *rad4-116* leading to eventual cell death.

## Conclusion

This study identified that the non-essential genes,*mrc1*, *swi1*, *swi3* and *hip1*, are required for survival under replication stress. The role these genes play in the resolution of DNA replication forks in combination with the loss of function from the hypomorphic *rad4-116*, appears to be the driving cause of the synthetic lethality of the double mutant strains. The inability of cells to recover from the fork stalling, and subsequent collapse caused cells to die during the depletion of the tested gene. In summary, this study provides the phenotypic analysis of strains to be able to identify non-essential genes required for survival under conditions of DNA stress. The work carried out in *S. pombe* could be built upon in higher eukaryotes to be able to map the interactions of TopBP1.

## Supplementary information

**Additional file 1: ** Supplemental Figure S1 showing the comparison the adjusted relative quantity of RNA of strains carrying the *V5* alleles. The figure shows the gradual decrease through graphical representation of the adjusted relative quantity between all the (A) Hip1-V5, (B) Swi1-V5, (C) Swi3-V5 and (D) Mrc1-V5 strains across the 24 hour timecourse after the addition of thiamine to the media.

## Data Availability

All the data required for the processing of the conclusions are presented in the results section. Supporting data was included separately.

## Abbreviations

ATR: ATM and Rad3 related
BRCA: Breast cancer
BRCT: BRCA1 C-terminal domain
Cdc: Cell division cycle
CDK: Cyclin-dependent kinase
CHK1: Checkpoint 1
DAPI: 4’,6-diamino-2-phenyl-indole
DDK: Dbf4p-dependent kinase
DNA: Deoxyribonucleic acid
EMM: Edinburgh minimal medium
FACS: Fluorescent-activated cell sorting
G: Guanine
G1: Gap/growth phase 1
G2: Gap/growth phase 2
G2/M: The growth phase 2/mitosis boundary of the cell cycle
G418: Geneticin
GFP: Green fluorescent protein
HIRA: Histone cell cycle regulation defective homologue A
HU: Hydroxyurea
IR: Ionising radiation
Kan: Kanamycin
Leu: Leucine
M phase: Mitosis
MCM: Mini-chromosome maintenance
mRNA: Messenger ribonucleic acid
NAT: Nourseothricin
OD: Optical density measured
ORF: Open reading frame
ORC: Origin recognition complex
PCNA: Proliferating cell nuclear antigen
PCR: Polymerase chain reaction
Pol: Polymerase
PVDF: Polyvinylidene difluoride
Rad: Radiation
RFC: Replication factor complex
RNA: Ribonucleic acid
RPA: Replication protein A
RT-QPCR: Reverse transcriptase quantitative PCR
S. cerevisiae: Saccharomyces cerevisiae
S. pombe: Schizosaccharomyces pombe
S-phase: Synthesis phase
SDS: Sodium dodecyl sulphate
SDS-PAGE: Sodium dodecyl sulphate polyacrylamide gel electrophoresis
ssDNA: Single strand DNA
SWI/SNF: Switch/sucrose nonfermentable
T: Thymine
Top: Topoisomerase
Ura: Uracil
ura4: Gene encoding dihydroorotase (involved in the de novo biosynthesis of pyrimidines)
v/v: Volume for volume
wt: Wild-type
YES: Yeast extract with supplements
