# Supplementary figures and images for: Characterisation of unessential genes required for survival under conditions of DNA stress
Source: J Genet Eng Biotechnol. 2020 May 6;18:14. doi: 10.1186/s43141-020-00025-x (PMC7201005; doi:10.1186/s43141-020-00025-x)

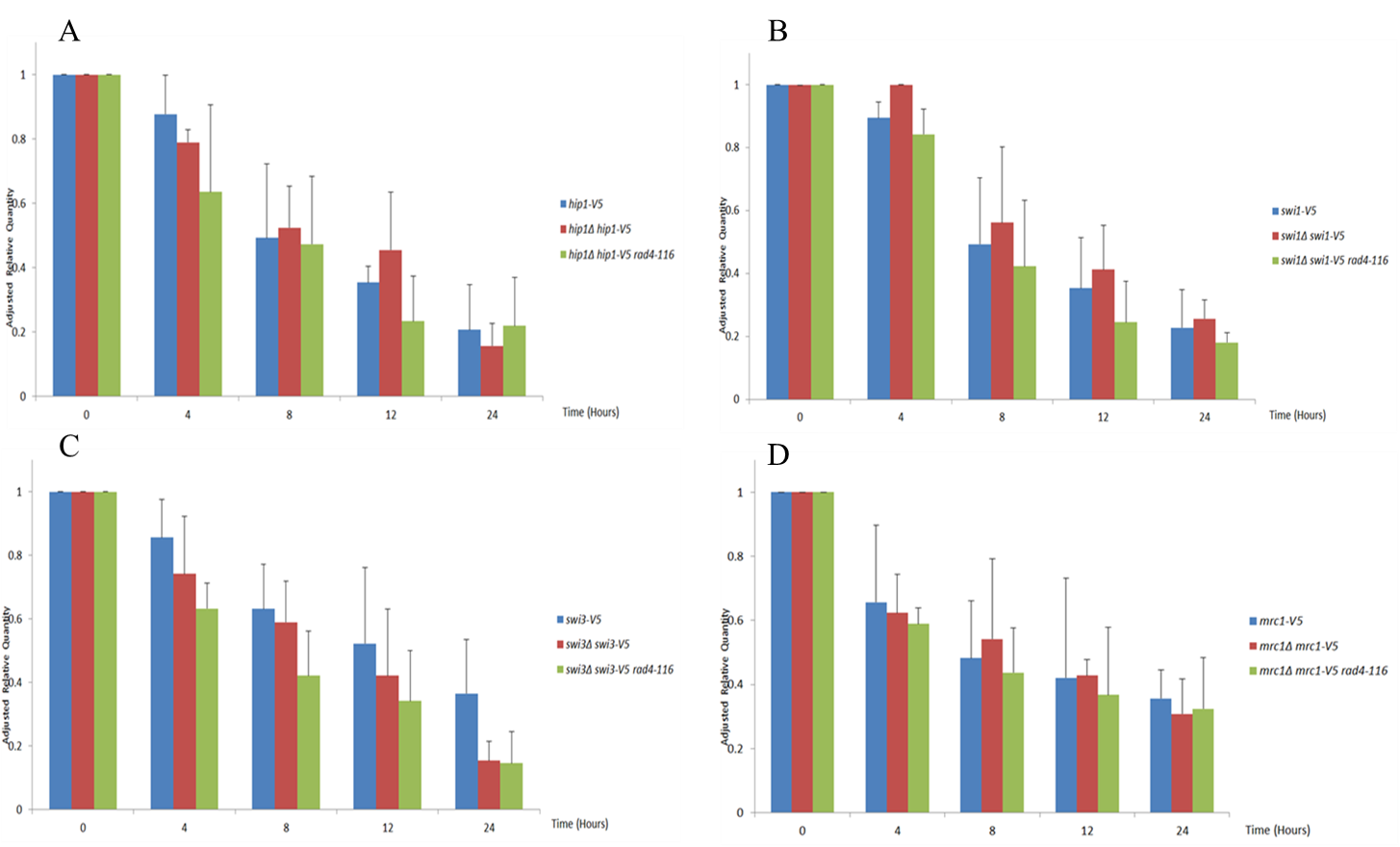

Supplement: Supplementary file 1 — Additional file 1: Supplemental Figure S1 showing the comparison the adjusted relative quantity of RNA of strains carrying the V5 alleles. The figure shows the gradual decrease through graphical representation of the adjusted relative quantity between all the (A) Hip1-V5, (B) Swi1-V5, (C) Swi3-V5 and (D) Mrc1-V5 strains across the 24 hour timecourse after the addition of thiamine to the media. [file 43141_2020_25_MOESM1_ESM.tif]
